# Dominant T cell receptor clonotypes in adrenocorticotropic hormone-secreting pituitary carcinoma are the highest-frequency clones among CD4^+^ and CD8^+^ cells in peripheral blood during effective anti-PD-1 therapy

**DOI:** 10.3389/fimmu.2026.1876390

**Published:** 2026-06-15

**Authors:** Mariko Sugiyama, Shintaro Iwama, Kazuhito Takeuchi, Tomoko Kobayashi, Takanori Murase, Daisuke Hagiwara, Hao Chen, Yuichi Nagata, Ryuta Saito, Yoshiki Akatsuka, Hiroshi Arima

**Affiliations:** 1 Department of Endocrinology and Diabetes, Nagoya University Graduate School of Medicine, Nagoya, Japan; 2 Department of Neurosurgery, Nagoya University Graduate School of Medicine, Nagoya, Japan; 3 Department of Immunology, Nagoya University Graduate School of Medicine, Nagoya, Japan

**Keywords:** Cushing’s syndrome, immunotherapy, PD-1, pituitary cancer, T−cell receptor repertoire

## Abstract

**Context:**

Pituitary carcinoma is a rare and highly aggressive tumor. While anti-programmed cell death-1 (PD-1) therapy has shown efficacy in some cases, the factors that predict a favorable response remain largely unclear.

**Objective:**

To evaluate tumor-infiltrating lymphocytes (TILs) in pituitary carcinoma and to compare T−cell receptor (TCR) clonotypes between the pituitary and peripheral blood.

**Methods:**

A 34-year-old woman with Lynch syndrome and adrenocorticotropic hormone-secreting pituitary carcinoma with hepatic metastasis received anti-PD-1 therapy, achieving durable disease control exceeding 1 year. Immunohistochemistry was performed on treatment-naïve surgical tumor samples, and TCR repertoire analyses were conducted on both the tumor sample and peripheral blood mononuclear cells collected during effective anti-PD−1 therapy.

**Results:**

Treatment-naïve pituitary carcinoma tissues exhibited infiltration of CD4^+^ and CD8^+^ T cells. Analysis of the TCR repertoire identified 15 clonotypes with a high frequency (> 1% of sequencing reads) in the tumor; among these, four of the five most prevalent clonotypes were co-detected as dominant clones in peripheral blood after treatment, including the most abundant clones found within the CD4^+^ and CD8^+^ T cell populations. Despite control of the primary and hepatic lesions, ovarian metastasis developed, which was associated with reduced CD4^+^ TILs.

**Conclusions:**

The presence of CD4^+^ and CD8^+^ TILs may underlie the immunological foundation for PD-1 blockade efficacy in pituitary carcinoma, supported by the detection of tumor-resident TCR clonotypes in peripheral blood during a positive therapeutic response.

## Introduction

Pituitary carcinoma is a rare (accounting for only 0.1–0.5% of all pituitary tumors) and highly aggressive malignancy defined by the presence of distant metastases (1). It has also been reported in patients with Lynch syndrome, a hereditary disorder resulting from germline mutations in mismatch repair (MMR) genes (2). Most pituitary carcinomas arise from functioning corticotroph or lactotroph lineages, and their clinical management remains challenging (1).

Temozolomide is the recommended first−line chemotherapy for pituitary carcinoma; however, the response rate is modest at approximately 40% (3), and most patients ultimately experience disease progression (1). Immune checkpoint inhibitors (ICIs), particularly programmed cell death 1 (PD-1) blockade, have emerged as a promising therapeutic option following temozolomide failure (3). Pembrolizumab, an anti-PD-1 antibody, has demonstrated clinical benefit in a subset of patients with pituitary carcinoma, but the therapeutic responses vary widely (4), and biomarkers predicting therapeutic response have yet to be established.

Recent advances in cancer immunotherapy highlight the pivotal role of tumor-infiltrating lymphocytes (TILs) and the clonality of the T cell receptor (TCR) repertoire in determining the response to immune checkpoint blockade in various solid tumors (5). Despite accumulating case reports of ICI use in pituitary carcinoma (4, 6–13), the immunological landscape remains poorly characterized. To date, intratumoral T cell infiltration has been addressed in only three reported cases, which relied exclusively on either hematoxylin and eosin staining (4) or post-radiotherapy samples (12). Furthermore, the characteristics of TCR clonotypes in pituitary carcinoma tissue and their relationship with circulating T cells during ICI therapy have been largely unexplored.

Here, we present a case of adrenocorticotropic hormone (ACTH)-secreting pituitary carcinoma with hepatic metastasis, in which we performed immunohistochemical analyses to characterize baseline TILs, as well as TCR repertoire profiling to compare clonotypes between the tumor and peripheral blood during PD−1 blockade.

## Materials and methods

### Study design and ethics

This single−patient analysis evaluated the immune characteristics of ACTH−producing pituitary carcinoma with distant metastasis in the context of PD−1 blockade. All procedures were approved by the Ethics Committee of Nagoya University Hospital (approval number 2018-0343), and signed informed consent was obtained directly from the patient.

### Sample collection

A pre−treatment pituitary tumor sample was obtained surgically prior to any anti−tumor therapy, including temozolomide or pembrolizumab, and was stored at −80 °C. Serum samples for anti-pituitary antibodies (APAs) analysis were also collected before pembrolizumab therapy and stored at −80 °C. Peripheral blood was collected during pembrolizumab therapy. Specifically, the peripheral blood sample for TCR repertoire analysis was collected in April 2024, after the 11^th^ cycle of pembrolizumab therapy, which was approximately 8 months after pembrolizumab initiation and the time point corresponding to clinical improvement. Peripheral blood mononuclear cells (PBMCs) were subsequently isolated from the blood samples and stored in liquid nitrogen.

### Histological and immunohistochemical analyses

Formalin−fixed, paraffin−embedded tumor sections were used for the histological andimmunohistochemical analyses. Histological analyses were performed as described previously (14). The primary antibodies used for immunohistochemistry are shown in **Supplementary Table 1**.

### TCR repertoire analysis

TCR β−chain repertoires were profiled by Repertoire Genesis Inc. (Osaka, Japan). The pituitary tumor sample used for TCR repertoire analysis was a fresh frozen sample, stored at −80 °C. RNA was extracted from the frozen tumor before ICI treatment and from magnetically isolated CD4^+^ and CD8^+^ T cells obtained from PBMCs after ICI treatment. The provider generated cDNA libraries from the RNA and analyzed the libraries using next-generation sequencing according to their standard protocol (15). The RNA input amounts were 465 ng for CD4^+^ T cells, 649 ng for CD8^+^ T cells, and 5 μg for pituitary carcinoma tissue. Clonotypes were defined based on identical complementarity-determining region 3 (CDR3) amino acid sequences and V/J gene usage, independent of nucleotide sequence identity. This approach accounts for the known phenomenon of convergent recombination, in which distinct nucleotide sequences encode identical CDR3 amino acid sequences, and is consistent with standard practices for TCR repertoire analysis. Clonotypes representing > 1% of total productive reads in the tumor were considered high−frequency clonotypes. Shared clonotypes were identified by comparing bulk tumor RNA with sorted peripheral CD4^+^ or CD8^+^ T cell subsets, and their intratumoral origin was inferred based on their detection within the corresponding subsets.

### Human leukocyte antigens (HLAs) and APAs

HLAs were analyzed using a previously reported method (16, 17). APAs were measured in human pituitary sections, as reported previously (16).

## Results

### Case description

**Figure 1:**
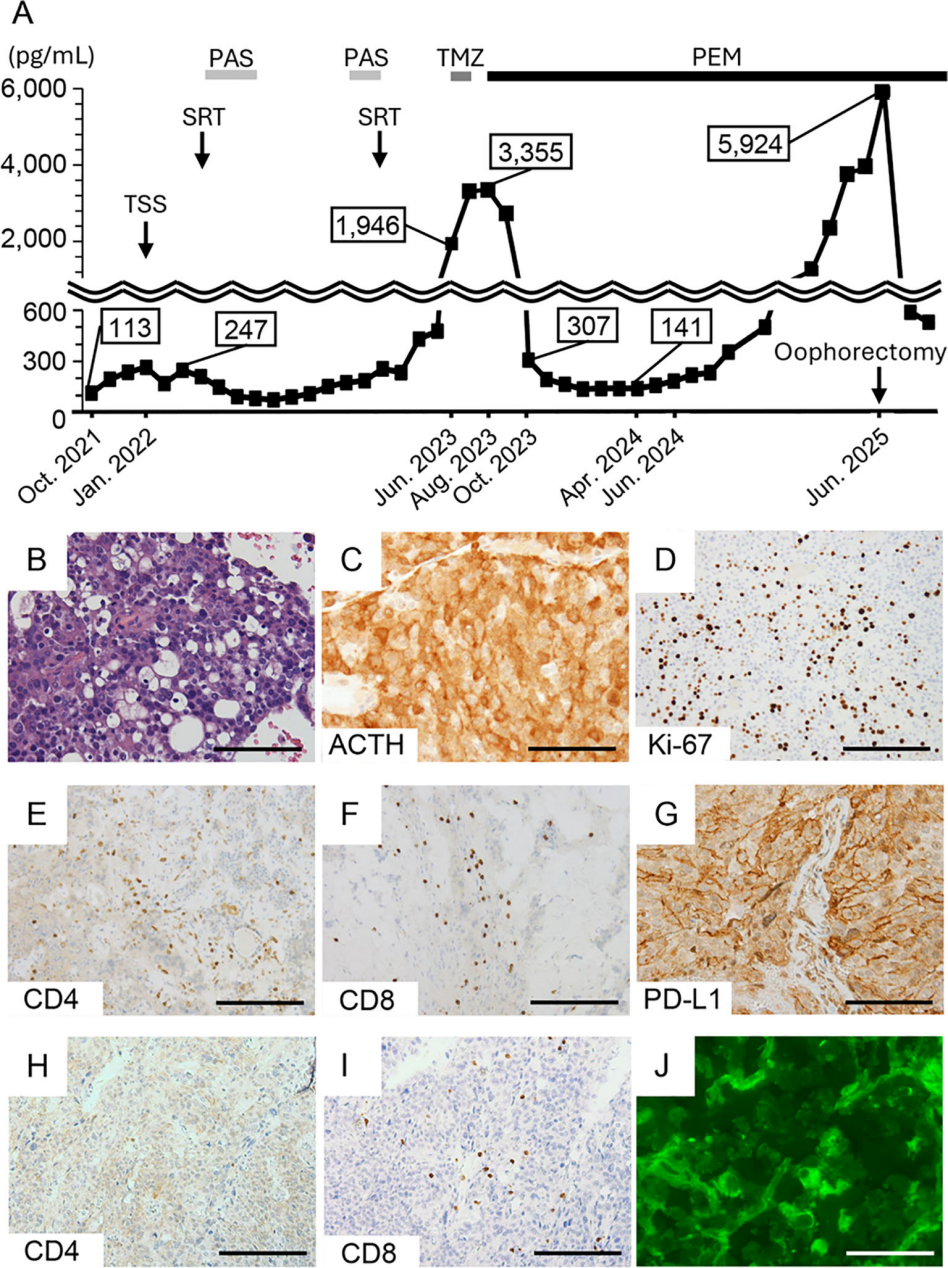
Clinical course and histopathological analyses. **(A)** Time course of the plasma ACTH level. **(B–G)** Histological [hematoxylin and eosin staining **(B)**] and immunohistochemical analyses [ACTH **(C)**, Ki-67 **(D)**, CD4 **(E)**, CD8 **(F)**, and PD−L1 **(G)** staining] in pituitary carcinoma tissue. **(H, I)** Immunohistochemical analyses of CD4 **(H)** and CD8 **(I)** in ovarian metastatic tissue. **(J)** Indirect immunofluorescence of anti−pituitary antibodies in normal human pituitary tissue and the patient’s serum; scale bar: 100 µm in B, C, and G and 200 µm in **(D–F, H–J)**; magnification: ×20 in **(B, C, G)** and ×10 in **(D–F, H–J)**. Abbreviations; TSS: transsphenoidal surgery, SRT: Stereotactic radiotherapy, PAS: pasireotide, TMZ: temozolomide, PEM: pembrolizumab.

In October 2021, a 34-year-old female with hypertension, diabetes, elevated ACTH and cortisol levels, and Cushingoid features including moon face and red striae was referred to our institute. She had undergone resection of sigmoid colon cancer at the age of 33 years and was diagnosed with Lynch syndrome due to a heterozygous mutation in the mismatch repair protein (MSH) 2 gene. Her morning ACTH level was 113 pg/mL (**Figure 1A**). The cortisol level was 26.5 µg/dL in the morning and 22.6 µg/dL at 23:00, while the 24−hour urinary free cortisol level was 1,900 µg/day. Low− (0.5 mg) and high−dose (8 mg) dexamethasone failed to suppress the cortisol. Inferior petrosal sinus sampling showed a central−to−peripheral ACTH ratio > 3. MRI revealed a 23 × 14 × 13 mm sellar mass with extension into the right cavernous sinus, suprasellar region, and sphenoid sinus.

In January 2022, endoscopic transsphenoidal surgery confirmed an ACTH−producing pituitary tumor with a Ki−67 index > 20%, nuclear atypia, and mitoses (**Figures 1B–D**). The postoperative ACTH level initially decreased but then rose to 247 pg/mL at 2 months, and residual tumor in the cavernous sinus was detected. Stereotactic radiotherapy and pasireotide 10 mg (every 4 weeks) were started. Immunohistochemistry revealed no somatostatin receptor (SSTR) 2A or SSTR5 expression, high microsatellite instability, and loss of MSH2/MSH6 expression in the tumor (**Supplementary Figures 1A, B**). Despite further stereotactic radiotherapy and pasireotide treatment, the tumor progressed, and metastasis appeared in the liver; the ACTH level rose to 1,946 pg/mL by June 2023 (**Figure 1A**). Although expression of O-6-methylguanine-DNA methyltransferase was absent in the tumor, temozolomide was ineffective. Subsequently, in August 2023, pembrolizumab 100 mg (every 3 weeks) was initiated. In October 2023, pembrolizumab treatment resulted in a rapid decrease in the ACTH level (from 3,355 pg/mL to 307 pg/mL), shrinkage of the hepatic metastases, and durable disease control for over 1 year with partial improvement of the Cushingoid features. Peripheral blood was collected for TCR repertoire analysis in April 2024, after the 11th cycle of pembrolizumab therapy. At that time, the plasma ACTH level was 141 pg/mL, the serum cortisol level was 0.1 µg/dL while receiving osilodrostat (6 mg/day) for hypercortisolism and supplemental dexamethasone (1 mg/day), and the absolute lymphocyte count was 2.6 × 10^3^/µL. Although pembrolizumab therapy had initially maintained disease control, the ACTH level gradually increased (up to 5,924 pg/mL by June 2025) together with the appearance of a new mass in the ovary. Oophorectomy and positive immunohistochemical staining for ACTH in the ovarian mass confirmed metastatic pituitary carcinoma, indicating secondary resistance to pembrolizumab.

### Immunological analyses

Pre−treatment pituitary carcinoma tissue showed CD4^+^ and CD8^+^ TILs (**Figures 1E, F**) with sparse CD20^+^ B cells (**Supplementary Figure 1C**). Programmed cell death-1 ligand-1 (PD-L1) expression was detectable on tumor cell surfaces (**Figure 1G**). Compared with the primary tumor, the ovarian lesion exhibited rare CD4^+^ TILs (**Figure 1H**) but a similar level of CD8^+^ TILs (**Figure 1I**). APAs were positive before pembrolizumab initiation (**Figure 1J**). The HLA haplotype of this patient was determined to be A*11:01-B*54:01-C*01:02-DRB1*04:05-DQB1*04:01-DPB1*05:01 and A*24:02-B*52:01-C*12:02-DRB1*15:01-DQB1*06:02-DPB1*05:01.

### TCR repertoire analysis

**Figure 2:**
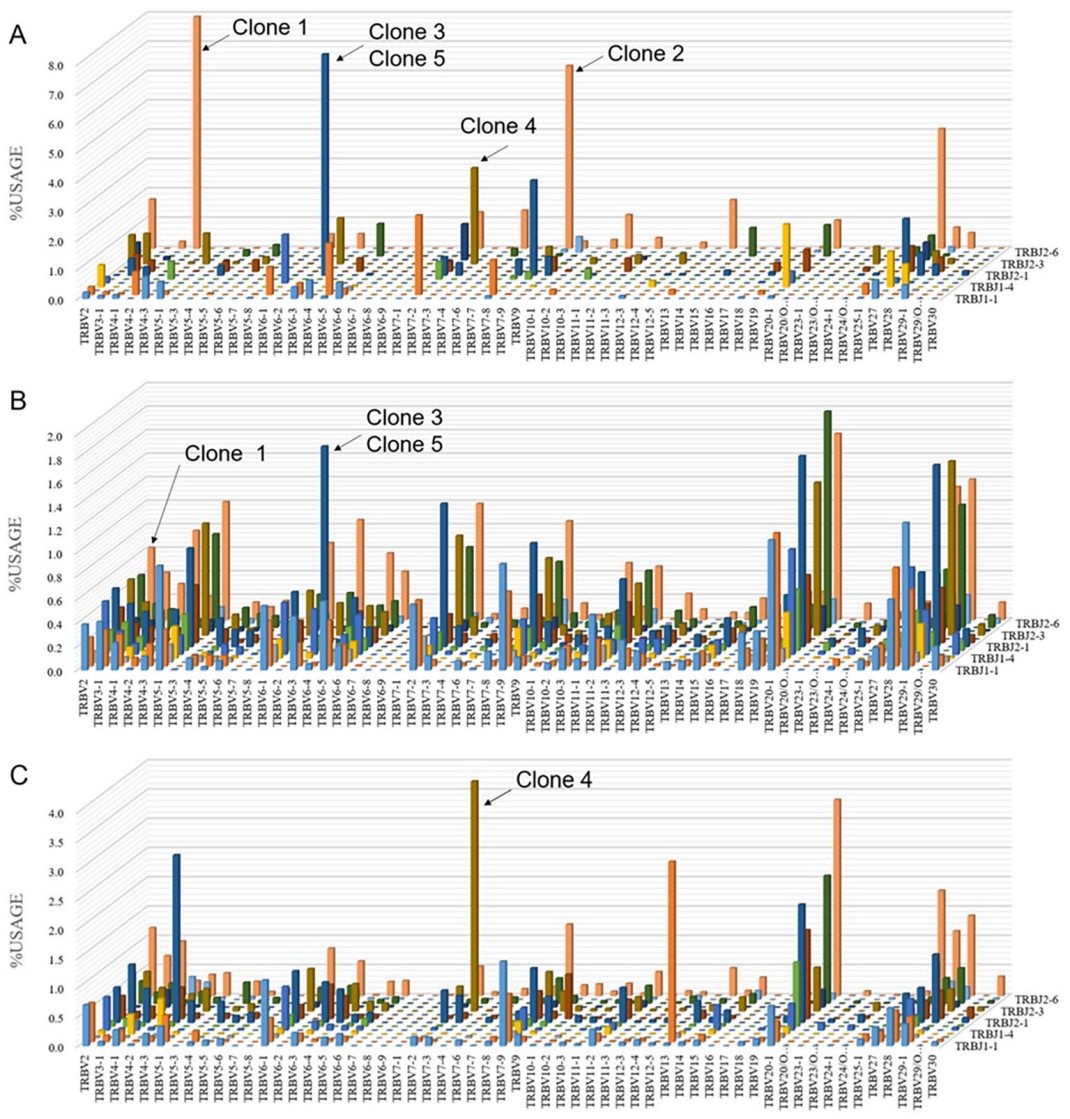
TCR repertoires. TCR repertoire profiles of CD8 TCRβ clonotypes identified in **(A)** pituitary carcinoma tissue, **(B)** CD4^+^ T cells from peripheral blood mononuclear cells (PBMCs), and **(C)** CD8^+^ T cells from PBMCs. The labels Clones 1–5 indicate the top five TCR clonotypes detected in the pituitary carcinoma tissue.

Tumor tissue before ICI treatment contained 15 high−frequency TCR clonotypes (> 1% reads; **Figure 2A**; **Supplementary Table 2**). The clonality metrics for the tumor and PBMC samples are summarized in the footnotes of **Supplementary Table 2**–**S4**. Among these 15 clonotypes, four of the five most prevalent clonotypes (ranks 1, 3, 4, and 5 in the tumor) were also detected as dominant clones in PBMCs after ICI treatment. Specifically, in CD4^+^ T cells, these appeared as rank 2 (corresponding to rank 1 in the tumor), rank 1 (corresponding to rank 3 in the tumor), and rank 4 (corresponding to rank 5 in the tumor) (**Figure 2B**; **Supplementary Table 3**); in CD8^+^ T cells, the corresponding clone was detected as rank 1 (corresponding to rank 4 in the tumor) (**Figure 2C**; **Supplementary Table 4**).

## Discussion

In this single-patient report, we demonstrated that (i) pituitary carcinoma tissue before any treatments harbored substantial CD4^+^ and CD8^+^ TILs; (ii) multiple tumor−dominant TCR clonotypes identified in the tumor before treatment were among the most abundant circulating CD4^+^ and CD8^+^ clones observed upon clinical improvement after PD−1 blockade; and (iii) in contrast, the ovarian lesion, which was refractory to PD−1 blockade, showed fewer CD4^+^ TILs. These findings suggest that tumor-resident TCR clonotypes are detectable in peripheral blood, potentially indicating an anti-tumor immune response.

Clinical treatment of pituitary carcinoma with ICIs remains limited to small series and case reports with heterogeneous responses (3). Majd et al. reported four patients treated with pembrolizumab (4). Of the three patients with corticotroph carcinomas, two achieved a partial response, and one exhibited stable disease for 4 months; the final patient, who had prolactin-secreting carcinoma, showed no objective response. This variability highlights both the therapeutic potential of ICIs and the urgent need for biomarkers beyond baseline clinicopathologic features. Notably, immune characterization of pituitary carcinoma tissue before ICI treatment has rarely been reported; of the two available studies, one employed only hematoxylin and eosin staining (4), and the other performed immunohistochemistry exclusively on tissues resected after prior radiation therapy (12). Our study demonstrated the presence of shared dominant TCR clonotypes in tumor tissue and peripheral blood during clinical improvement after ICI therapy, suggesting an important role of these clones in antitumor immunity.

ICIs reshape clonal dynamics by promoting the expansion and tumor infiltration of tumor−reactive T cell clonotypes, in parallel with development of a clinical response (5). Consistent with this mechanism, studies in solid tumors, particularly melanoma treated with anti−PD−1 therapy, have shown that PD−1 blockade induces marked changes in TCR repertoire and clonal behaviors indicative of clinically meaningful antitumor immunity (18). In renal cell carcinoma and lung cancer, intratumoral TCR clonal expansion is correlated with therapeutic efficacy, whereas peripheral blood TCR clonal changes do not always mirror intratumoral alterations (19, 20). Our results suggest that overlap of dominant TCR clonotypes between pre-ICI TILs and post-ICI PBMCs may represent a hallmark of the treatment response in pituitary carcinoma.

Pembrolizumab is approved for tumors exhibiting high MSI or MMR deficiency. In the aforementioned study by Majd et al., one of the patients with a partial response exhibited a hypermutator phenotype with MSH2/MSH6 mutations detected in tumor tissue post-treatment with an alkylating agent. To our knowledge, the patient in this study is the first to demonstrate ICI responsiveness of pituitary carcinoma arising in the context of Lynch syndrome. Taken together, MMR deficiency/hypermutation−related features may contribute to a benefit in at least a subset of pituitary carcinomas.

Despite durable control of the pituitary and liver lesions, the development of ovarian metastasis indicated spatial heterogeneity and possible site−specific immune escape during PD−1 blockade. Spatially heterogeneous responses to ICIs are becoming increasingly recognized and may carry prognostic implications differing from those of a uniform response (21). Resistance to ICI blockade may arise through diverse tumor−intrinsic and microenvironmental mechanisms (22). CD4^+^ T cells play pivotal roles in orchestrating effective antitumor immunity by supporting CD8^+^ T cell responses (23). In our patient, the scarcity of CD4^+^ TILs in the ovarian lesion may have compromised the antitumor immune activity elicited by PD-1 blockade. In addition, the ovary is an immune-privileged organ, which may explain the generally limited efficacy of ICIs in ovarian cancer (24). However, these interpretations are descriptive and preliminary; further studies are essential to clarify the precise mechanisms underlying this site-specific immune escape.

The patient’s HLA background is noteworthy, as host HLA alleles can influence antigen presentation and have been implicated in the susceptibility to ICI-related pituitary immune−related adverse events, including ACTH deficiency (25, 26). The HLA−DRB1*15:01–DQB1*06:02 haplotype identified in this patient is associated with ICI-induced pituitary dysfunction (17), and the presence of pretreatment APAs—a known risk factor for ICI-related pituitary dysfunction (16)—suggests a potential predisposition to pituitary-directed autoimmunity during ICI blockade. These observations suggest that immune mechanisms underlying ICI-induced ACTH deficiency intersect with the antitumor response in this ACTH−secreting pituitary carcinoma.

This study has limitations. First, as a single-patient analysis, our findings have limited generalizability and thus should be interpreted with caution. Second, the lack of available pretreatment PBMC samples for TCR sequencing prevented distinguishing therapy-induced clonal expansion from pre-existing circulating clones. Third, TCR repertoire dynamics were assessed at a single time point; serial sampling is required to clarify the relationship between clonal expansion and clinical response. Fourth, the antigen specificity of the dominant T cell clonotypes remains unclear; therefore, we could not definitively conclude that these T cells are specific to tumor-associated antigens. Given the presence of pre-treatment APAs, some of these dominant clones might represent autoimmune rather than tumor-reactive T cells. Fifth, immune microenvironment characterization was limited to IHC and did not include advanced spatial or immune subset analyses using techniques such as multiplex immunofluorescence or single-cell RNA sequencing. In addition, the use of a non-clinically standardized PD-L1 antibody limited the reliability of PD-L1 expression interpretation. Finally, the lack of TCR profiling in the ovarian metastatic lesion limits our mechanistic understanding of resistance and spatial heterogeneity. As a methodological limitation, tumor TCRβ profiling was performed using bulk tumor RNA without CD4^+^/CD8^+^ T-cell sorting; therefore, intratumoral T-cell subset assignments were inferred solely based on detection in the corresponding peripheral blood subsets. Future studies using *in situ* profiling or sorted TILs are warranted to provide more definitive evidence.

In conclusion, our findings from this case of ACTH−secreting pituitary carcinoma associated with Lynch syndrome suggest that the presence of CD4^+^ and CD8^+^ TILs prior to PD-1 blockade, as well as the detection of identical TCR clonotypes as dominant clones in the peripheral blood during effective treatment, provide a potential immunological basis for treatment efficacy. Since the results were inherently limited by the single-case design, further studies involving larger cohorts are warranted to validate these observations and determine their broader clinical implications.

## Data Availability

The original contributions presented in the study are included in the article/**Supplementary Material**. Further inquiries can be directed to the corresponding authors.

## Supplementary material

Supplementary Figure 1 Histopathological analyses. **(A–C)** Immunohistochemical analyses of MSH2 **(A)**, MSH6 **(B)**, and CD20 **(C)** in pituitary carcinoma tissue; scale bar: 200 µm in **(A–C)**; magnification: ×10 in **(A–C)**.

Supplementary Table 1 Antibodies used for immunohistochemistry and immunofluorescence staining.

Supplementary Table 2 Top 20 T cell clones identified in pituitary carcinoma tissue.

Supplementary Table 3 Top 10 T cell clones identified in CD4^+^ T cells from peripheral blood mononuclear cells.

Supplementary Table 4 Top 10 T cell clones identified in CD8+ T cells from peripheral blood mononuclear cells.
