# Supplementary figures and images for: Dominant T cell receptor clonotypes in adrenocorticotropic hormone-secreting pituitary carcinoma are the highest-frequency clones among CD4+ and CD8+ cells in peripheral blood during effective anti-PD-1 therapy
Source: Front Immunol. 2026 Jun 15;17:1876390. doi: 10.3389/fimmu.2026.1876390 (PMC13311078; doi:10.3389/fimmu.2026.1876390)

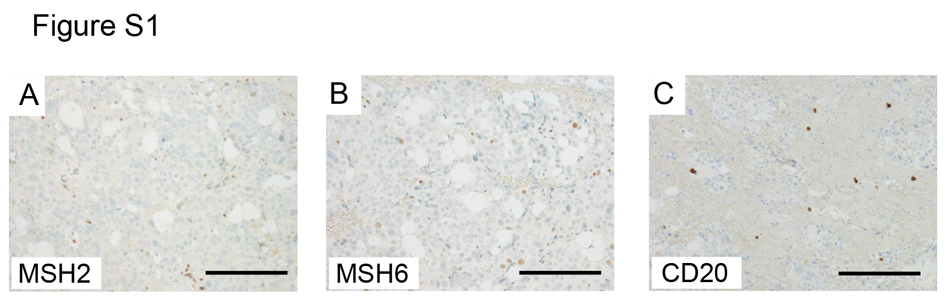

Supplement: Supplementary Figure 1 — Histopathological analyses. (A–C) Immunohistochemical analyses of MSH2 (A), MSH6 (B), and CD20 (C) in pituitary carcinoma tissue; scale bar: 200 µm in (A–C); magnification: ×10 in (A–C). [file Image1.tif]
