# Supplementary material for: Dominant T cell receptor clonotypes in adrenocorticotropic hormone-secreting pituitary carcinoma are the highest-frequency clones among CD4+ and CD8+ cells in peripheral blood during effective anti-PD-1 therapy
Source: Front Immunol. 2026 Jun 15;17:1876390. doi: 10.3389/fimmu.2026.1876390 (PMC13311078; doi:10.3389/fimmu.2026.1876390)
Supplement: Supplementary Table 1 — Antibodies used for immunohistochemistry and immunofluorescence staining. [file Table1.doc]

**Supplementary Table 1.** **Antibodies used for immunohistochemistry and immunofluorescence staining.**

| Primary antibody | ID | Company | Applications |
| --- | --- | --- | --- |
| anti-CD4 | Clone ID: SP35 | Roche Diagnostics | IHC |
| anti-CD8 | Clone ID: SP57 and SP239 | Roche Diagnostics | IHC |
| anti-CD20 | Clone ID: SP32 | Abcam | IHC |
| anti-PD-L1 | Catalog ID: 19952-1-AP | Proteintech | IHC |
| anti-CD4 | Clone ID: SK3 | BioLegend | Cell sorting |
| anti-CD8 | Clone ID: RPA-T8 | BioLegend | Cell sorting |

**Abbreviations:** ACTH, adrenocorticotropic hormone; PD-L1, programmed cell death-1 ligand 1; IHC, immunohistochemistry.
