# Supplementary material for: Dominant T cell receptor clonotypes in adrenocorticotropic hormone-secreting pituitary carcinoma are the highest-frequency clones among CD4+ and CD8+ cells in peripheral blood during effective anti-PD-1 therapy
Source: Front Immunol. 2026 Jun 15;17:1876390. doi: 10.3389/fimmu.2026.1876390 (PMC13311078; doi:10.3389/fimmu.2026.1876390)
Supplement: Supplementary Table 2 — Top 20 T cell clones identified in pituitary carcinoma tissue. [file Table2.doc]

**Supplementary Table 2. Top 20 T cell clones identified in pituitary carcinoma tissue.**

| Rank | TRBV | TRBJ | CDR3 | Reads | %Reads | Clone ID |
| --- | --- | --- | --- | --- | --- | --- |
| 1 | TRBV4-2 | TRBJ2-7 | CASSQDLGGWREQYF | 2,057 | 6.70 | 1 |
| 2 | TRBV7-9 | TRBJ2-7 | CASSFGPSGSARDEQYF | 1,658 | 5.40 | 2 |
| 3 | TRBV6-3 | TRBJ2-1 | CASKAGYNEQFF | 1,225 | 3.99 | 3 |
| 4 | TRBV7-3 | TRBJ2-3 | CASSFDSGGTDTQYF | 910 | 2.96 | 4 |
| 5 | TRBV6-3 | TRBJ2-1 | CASKDGYNEQFF | 745 | 2.43 | 5 |
| 6 | TRBV27 | TRBJ2-7 | CASSNDIHEQYF | 724 | 2.36 |  |
| 7 | TRBV7-2 | TRBJ1-2 | CASSSLRTGQNYGYTF | 597 | 1.94 |  |
| 8 | TRBV7-9 | TRBJ2-1 | CASSLWGIATSGGPAYNEQFF | 564 | 1.84 |  |
| 9 | TRBV6-5 | TRBJ1-2 | CASKWEGGYGYTF | 489 | 1.59 |  |
| 10 | TRBV20-1 | TRBJ1-4 | CSAPDREAEKLFF | 480 | 1.56 |  |
| 11 | TRBV13 | TRBJ2-7 | CASSQQGADEQYF | 477 | 1.55 |  |
| 12 | TRBV7-6 | TRBJ2-7 | CASSLGEVREQYF | 365 | 1.19 |  |
| 13 | TRBV27 | TRBJ1-4 | CASSPGQGRANEKLFF | 345 | 1.12 |  |
| 14 | TRBV7-8 | TRBJ1-2 | CASTGDSRGFTF | 339 | 1.10 |  |
| 15 | TRBV6-3 | TRBJ2-3 | CASSYSRHTDTQYF | 317 | 1.03 |  |
| 16 | TRBV6-1 | TRBJ1-5 | CASETVNNQPQHF | 301 | 0.98 |  |
| 17 | TRBV10-3 | TRBJ2-7 | CAISERAGGDEQYF | 288 | 0.94 |  |
| 18 | TRBV27 | TRBJ2-7 | CASSIRDRGYEQYF | 282 | 0.92 |  |
| 19 | TRBV2 | TRBJ2-3 | CASRPSGSSADTQYF | 276 | 0.90 |  |
| 20 | TRBV6-5 | TRBJ2-5 | CASSYSGVSLAETQYF | 272 | 0.89 |  |

Sequencing depth: 277,487 total reads; number of productive reads: 30,697; clonality metrics: Shannon–Weaver index (H') = 5.273, Inverse Simpson index (1/λ) = 64.902, and Pielou's evenness = 0.669; normalization strategy: clonotype frequencies were normalized to the total number of productive reads; dominant clones were operationally defined as the highest-frequency clonotypes within the pituitary carcinoma tissue T-cell repertoire.

**Abbreviations:** TRBV, T cell receptor β chain V gene; TRBJ, T cell receptor β chain J gene; CDR3, complementarity-determining region 3.
