# Supplementary material for: Dominant T cell receptor clonotypes in adrenocorticotropic hormone-secreting pituitary carcinoma are the highest-frequency clones among CD4+ and CD8+ cells in peripheral blood during effective anti-PD-1 therapy
Source: Front Immunol. 2026 Jun 15;17:1876390. doi: 10.3389/fimmu.2026.1876390 (PMC13311078; doi:10.3389/fimmu.2026.1876390)
Supplement: Supplementary Table 3 — Top 10 T cell clones identified in CD4+ T cells from peripheral blood mononuclear cells. [file Table3.doc]

**Supplementary Table 3. Top 10 T cell clones identified in CD4^+^ T cells from peripheral blood mononuclear cells.**

| Rank | TRBV | TRBJ | CDR3 | Reads | %Reads | Clone ID |
| --- | --- | --- | --- | --- | --- | --- |
| 1 | TRBV6-3 | TRBJ2-1 | CASKAGYNEQFF | 2,511 | 0.76 | 3 |
| 2 | TRBV4-2 | TRBJ2-7 | CASSQDLGGWREQYF | 1,972 | 0.60 | 1 |
| 3 | TRBV20-1 | TRBJ2-5 | CSARERETQYF | 851 | 0.26 |  |
| 4 | TRBV10-3 | TRBJ2-7 | CAISERAGGDEQYF | 615 | 0.19 | 5 |
| 5 | TRBV6-3 | TRBJ2-1 | CASKDGYNEQFF | 544 | 0.16 |  |
| 6 | TRBV29-1 | TRBJ2-3 | CSVDGQGGDTQYF | 486 | 0.15 |  |
| 7 | TRBV5-1 | TRBJ2-3 | CASSLGQGNGDTQYF | 459 | 0.14 |  |
| 8 | TRBV29-1 | TRBJ2-6 | CSVISGANVLTF | 450 | 0.14 |  |
| 9 | TRBV29-1 | TRBJ1-5 | CSVEDPPTTKNSNQPQHF | 310 | 0.09 |  |
| 10 | TRBV29-1 | TRBJ2-5 | CSVRRGQETQYF | 294 | 0.09 |  |

Sequencing depth: 464,937 total reads; number of productive reads: 330,793; clonality metrics: Shannon–Weaver index (H') = 9.569, Inverse Simpson index (1/λ) = 4,638.821, and Pielou's evenness = 0.890; normalization strategy: clonotype frequencies were normalized to the total number of productive reads; dominant clones were operationally defined as the highest-frequency clonotypes within the sorted CD4^+^ T cell population.

**Abbreviations:** TRBV, T cell receptor β chain V gene; TRBJ, T cell receptor β chain J gene; CDR3, complementarity-determining region 3.
