# Supplementary material for: Dominant T cell receptor clonotypes in adrenocorticotropic hormone-secreting pituitary carcinoma are the highest-frequency clones among CD4+ and CD8+ cells in peripheral blood during effective anti-PD-1 therapy
Source: Front Immunol. 2026 Jun 15;17:1876390. doi: 10.3389/fimmu.2026.1876390 (PMC13311078; doi:10.3389/fimmu.2026.1876390)
Supplement: Supplementary Table 4 — Top 10 T cell clones identified in CD8+ T cells from peripheral blood mononuclear cells. [file Table4.doc]

**Supplementary Table 4. Top 10 T cell clones identified in CD8+ T cells from peripheral blood mononuclear cells.**

| Rank | TRBV | TRBJ | CDR3 | Reads | %Reads | Clone ID |
| --- | --- | --- | --- | --- | --- | --- |
| 1 | TRBV7-3 | TRBJ2-3 | CASSFDSGGTDTQYF | 3,717 | 3.42 | 4 |
| 2 | TRBV13 | TRBJ1-2 | CASRFDSSEEGYTF | 3,042 | 2.80 |  |
| 3 | TRBV4-3 | TRBJ2-1 | CASRELGGGYNEQFF | 2,270 | 2.09 |  |
| 4 | TRBV20-1 | TRBJ2-2 | CSARLAGDTGELFF | 882 | 0.81 |  |
| 5 | TRBV10-1 | TRBJ2-2 | CASSESIPSTTNTGELFF | 755 | 0.69 |  |
| 6 | TRBV20-1 | TRBJ2-5 | CSAREEEGAIETQYF | 705 | 0.65 |  |
| 7 | TRBV7-9 | TRBJ1-1 | CASSPRTGGMNTEAFF | 621 | 0.57 |  |
| 8 | TRBV20-1 | TRBJ1-6 | CSASPGGLSPLHF | 593 | 0.55 |  |
| 9 | TRBV6-1 | TRBJ1-1 | CASSEEVINTEAFF | 578 | 0.53 |  |
| 10 | TRBV4-3 | TRBJ1-4 | CASSQGGGTGTEKLFF | 564 | 0.52 |  |

Sequencing depth: 149,169 total reads; number of productive reads: 108,784; clonality metrics: Shannon–Weaver index (H') = 8.237, Inverse Simpson index (1/λ) = 320.189, and Pielou's evenness = 0.839; normalization strategy: clonotype frequencies were normalized to the total number of productive reads; dominant clones were operationally defined as the highest-frequency clonotypes within the sorted CD8^+^ T cell population.

**Abbreviations:** TRBV, T cell receptor β chain V gene; TRBJ, T cell receptor β chain J gene; CDR3, complementarity-determining region 3.
